# A Web-Based Nutrition Program Reduces Health Care Costs in Employees With Cardiac Risk Factors: Before and After Cost Analysis

**DOI:** 10.2196/jmir.1263

**Published:** 2009-10-23

**Authors:** Naomi Sacks, Howard Cabral, Lewis E Kazis, Kelli M Jarrett, Delia Vetter, Russell Richmond, Thomas J Moore

**Affiliations:** ^4^Boston University School of MedicineBostonMAUSA; ^3^Verisk HealthIncBostonMAUSA; ^2^EMC CorporationBostonMAUSA; ^1^Boston University School of Public HealthBostonMAUSA

**Keywords:** Employer health costs, disease management, health promotion, wellness programs, costs and cost analysis

## Abstract

**Background:**

Rising health insurance premiums represent a rapidly increasing burden on employer-sponsors of health insurance and their employees. Some employers have become proactive in managing health care costs by providing tools to encourage employees to directly manage their health and prevent disease. One example of such a tool is DASH for Health, an Internet-based nutrition and exercise behavior modification program. This program was offered as a free, opt-in benefit to US-based employees of the EMC Corporation.

**Objective:**

The aim was to determine whether an employer-sponsored, Internet-based diet and exercise program has an effect on health care costs.

**Methods:**

There were 15,237 total employees and spouses who were included in our analyses, of whom 1967 enrolled in the DASH for Health program (DASH participants). Using a retrospective, quasi-experimental design, study year health care costs among DASH participants and non-participants were compared, controlling for baseline year costs, risk, and demographic variables. The relationship between how often a subject visited the DASH website and health care costs also was examined. These relationships were examined among all study subjects and among a subgroup of 735 subjects with cardiovascular conditions (diabetes, hypertension, hyperlipidemia). Multiple linear regression analysis examined the relationship of program use to health care costs, comparing study year costs among DASH participants and non-participants and then examining the effects of increased website use on health care costs. Analyses were repeated among the cardiovascular condition subgroups.

**Results:**

Overall, program use was not associated with changes in health care costs. However, among the cardiovascular risk study subjects, health care costs were US$827 lower, on average, during the study year (*P* *=* .05; *t*_729_ = 1.95). Among 1028 program users, increased website use was significantly associated with lower health care costs among those who visited the website at least nine times during the study year (US$14 decrease per visit; *P* = *.*04; *t*_1022_ = 2.05), with annual savings highest among 80 program users with targeted conditions (US$55 decrease per visit; *P* < .001; *t*_74_ = 2.71).

**Conclusions:**

An employer-sponsored, Internet-based diet and exercise program shows promise as a low-cost benefit that contributes to lower health care costs among persons at higher risk for above-average health care costs and utilization.

## Introduction

Health insurance premiums have risen faster than inflation for the past 10 years, placing an increasing burden on employer-sponsors of health insurance and their employees [1,2]. Some employers have become proactive in managing health costs, providing tools that encourage employees to directly manage their health and prevent disease [3]. Examples include smoking cessation and stress management programs, gym and health club memberships, and formal disease management programs, many of which are popular with employees and improve employee satisfaction. But there is very little evidence that any of these initiatives actually reduce health care costs [4].

Recent reviews of employer health promotion programs show some success in improving employee health and productivity, but show mixed results as to whether or not these programs have an impact on health care costs [5]. Employers commonly offer nutrition education programs [6,7], but there is little evidence that such programs alter eating behaviors or change health care costs. Yet rapidly rising rates of overweight and obesity can contribute to a number of high-cost chronic diseases (eg, diabetes, hypertension, heart disease), increasing the likelihood that health care costs for these already highly prevalent and expensive conditions will increase dramatically in the future.

**Figure 1:**
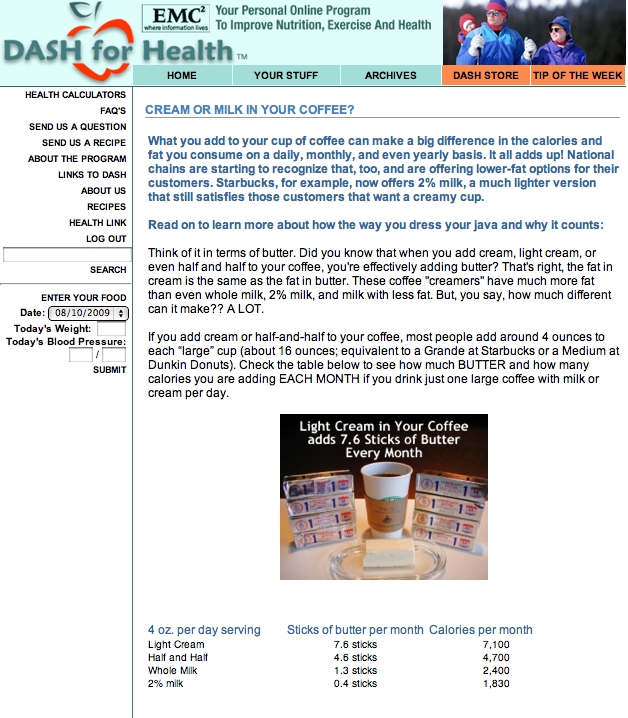
Example of article provided on the DASH for Health website

**Figure 2:**
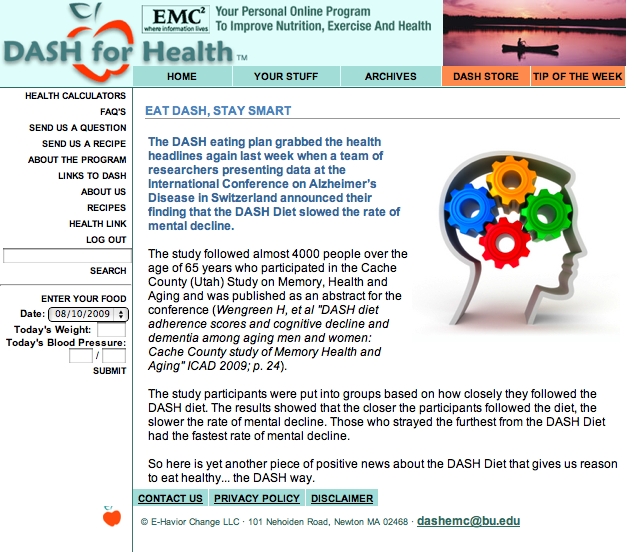
Example of article provided on the DASH for Health website

To address the issue of poor nutrition and the diseases associated with it, we designed an Internet-based nutrition and exercise behavior modification program called DASH for Health. Our program was based on the National Heart Lung and Blood Institute (NHLBI) DASH diet, which was originally developed to lower blood pressure and which has been demonstrated in randomized controlled trials to lower blood pressure and cholesterol levels and heighten insulin sensitivity [8-12]. We developed the Web-based DASH for Health program in collaboration with EMC Corporation, a Massachusetts-based global information infrastructure company. The program was offered as a free employee benefit to US-based EMC employees and their family members, who could opt-in to the program and were free to use the program however intensely they chose. Employees and their spouses were eligible to enroll in the program at the beginning of the calendar year in which it was offered. During the first year this program was offered at EMC Corporation (the same year that the health costs reported in this paper were collected), enrollees in the program were found to have significantly lost weight, lowered their blood pressure, and improved their healthy eating habits [11]. Examples of articles provided on the DASH for Health website are shown in Figure 1 and Figure 2.

To determine whether an Internet-based behavior modification program like DASH for Health has any effect on health care costs, we analyzed the costs for EMC employees and their spouses during the 12 months preceding the initial launch of the DASH for Health program and during the 12 months following the launch. The baseline year was the 12 months immediately preceding the initial launch of the program, and the study year was the 12 months immediately following the launch. We compared health care costs of those who participated in the DASH for Health program with those of nonparticipants. We analyzed costs for all study subjects and then performed a more focused analysis on employees and spouses with medical conditions (hypertension, hyperlipidemia, and/or diabetes) targeted by the DASH program.

## Methods

### Study Setting

 The DASH for Health website was developed by a team at Boston University School of Medicine (BUSM). The website provides tools for enrollees to record and follow their body weight, blood pressure, eating habits, and exercise habits as well as providing a variety of healthy recipes based on the DASH diet. These tools include easy-to-use entry fields for entering weight, blood pressure, 24-hour food recall, and exercise habits. Although enrollees were encouraged to use these data “progress reports” as a means of encouraging their adherence to the program, there were no requirements or set expectations of how often enrollees would enter their own data. The website also provides two new articles each week on improving nutritional habits (based on the DASH diet) or healthy exercise. Everyone who enrolls in the program gets a reminder email each time a new article is posted on the website. The website is hosted at SignalZ Corporation (Montpelier, VT, USA). Self-reported data on demographics (age, gender, and employee/dependent status) and website visits during each year were collected by SignalZ and transferred to the research team at BUSM. Because enrollees had to enter the site using their username and password, standard website monitoring software was used to track how many times a particular enrollee visited the site. We define a “visit” as a unique instance of an enrollee logging on to the site.

When the program was about to launch, EMC Corporation informed employees that DASH for Health would be available at no cost to them and their adult household members. EMC had no other role in the program. EMC management had no access to the identities of the subjects or to their medical data. Once the program began, the employer took no role in encouraging website usage. The announcing emails from EMC leadership clearly stated that the employer would not know any enrollee’s individual data or the identities of people who did and did not enroll.

EMC’s clinical data warehouse, Ingenix, provided demographic and medical and pharmacy cost data for employees and their adult dependents. These data included date of birth, gender, and employee status. D2Hawkeye, a medical analytics firm specializing in data warehousing and health claims analysis, provided data cleansing services and summarized study subjects’ demographic information, condition specific diagnoses, and total health care costs. D2Hawkeye calculated a risk adjustment factor for each study subject, using a proprietary methodology that combines diagnostic, procedural, and pharmacy information in health claims data with pattern recognition and times series analysis. The risk index provides a single representation of an individual’s disease burden and has been shown to correctly distinguish high-cost and low-cost persons 77% of the time. Additionally, the likelihood that an individual will be in a high-cost group increases as the risk index value increases. We used an extension of the risk index, the adjusted risk index (ARI), which incorporates assessments of gaps in care (medical or pharmaceutical) for various conditions [13].

We used individual subject-level linkages to merge data on DASH for Health participants’ website visit frequency with health care cost data and other variables. A subset of study subjects was identified with evidence of diabetes, hypertension, or hyperlipidemia, conditions that could be expected to benefit from the improved dietary behaviors that are encouraged by the DASH for Health program. This subset is referred to as the cardiovascular (CV) risk group. Data were managed and analyzed using SAS, version 9.1.3 (SAS Institute Inc, Cary, NC, USA). Approval for this study was obtained from the Boston University Institutional Review Board.

Study subjects with missing data for key fields such as age, employee status, or gender were excluded, as were DASH participants with no matching entries in the health care cost files. These enrollees were either not covered by the EMC insurance plan or were not affiliated with EMC at the end of the study period. The study was limited to study subjects with health care costs in both study years to ensure that those with bad claims data reported as negative or zero amounts were excluded from the analysis and to increase the likelihood that the analysis was restricted to those with primary insurance coverage through EMC. Information on job classification was not included since baseline year health care costs and risk assessment are proximal for our analysis.

The total number of EMC employees and their spouses was 29,675, of which approximately 75% (n = 22,354) were reported to have received health coverage through EMC and had health care costs in the baseline or study year. Sixty-eight percent (n = 15,237) of these subjects had costs in both years. Additionally, 3797 EMC employees or spouses enrolled in the DASH for Health program and used the DASH program website at least once in the study year (DASH participants). Seventy-three percent (n = 2756) of these DASH participants were found in the health claims summary file, and 71% (n = 1967) of these 2756 had health care costs in both years. The final study sample (N = 15,237, DASH = 1967, non-DASH = 13,270) also reflected the restrictions described above.

### Analysis

In univariate analyses to describe our sample, we generated means and standard deviations for continuous variables and counts with percentages for categorical variables. We used bivariate analyses to examine the relationships among demographic, annual health care cost, and website usage data for 15,237 study subjects over the 2-year baseline and study year period. In these analyses, we employed chi-square tests for analyses of categorical data and two-sample *t* tests for comparisons of continuous by categorical variables. Multiple linear regression analysis examined the relationship of DASH program enrollment with study year health care costs, controlling for other salient factors. To control for potential bias of self-selection of the DASH participants, we used a logistic regression model to construct a propensity score that represented the likelihood of participation in the DASH program. Studies that use observational data, as ours does, are subject to bias because the “treatment” and “control groups” are not randomly assigned and may differ in ways that affect the outcome of interest. A propensity score, which represents the conditional probability of receiving a given treatment, given a vector of measured covariates, is frequently used in such studies to adjust for differences in the observed characteristics between the treatment and the control groups. Our use of logistic regression is one of several recommended approaches for determining the propensity score [14].

All available study variables were included in the propensity score model (baseline year risk index and ARI, baseline year costs, age, gender, and employee status). We then used a linear regression model to compare study year costs among DASH and non-DASH participants, controlling for age, gender, ARI, baseline year costs, and likelihood of DASH participation (propensity score). In this model, study year total health care costs was the dependent (outcome) variable; independent (predictor) variables were DASH participation, age, gender, employee status, baseline year ARI, and baseline year total health care costs. Next, because stronger effects of an intervention are often observed in the most compliant or frequent participants, we examined whether more frequent use of the website than is typical is more strongly associated with reduced costs among DASH participants than across the full range of website use overall. For this, we used a similar linear regression analysis to evaluate the relationship of website usage intensity at or above the median to health care costs in the study year. Study year total health care costs was the dependent variable; independent variables were number of website visits, age, gender, employee status, baseline year ARI, and baseline year total health care costs. We repeated these analyses using the 735 DASH CV risk group and non-DASH CV risk group study subjects who showed evidence in both study years of diabetes, hyperlipidemia, or hypertension.

To address the skewed distribution of the cost data, baseline and study year costs in all analyses were top-coded at US$25,000, which represents the 99th percentile of annual health care costs in our study population. Website usage was top-coded at 75 website visits to address skewness. The analyses were repeated using trimming to remove high-cost outliers, using the entire study population and using top-coding at other thresholds. As top-coding is recommended for reducing the effects of high-cost outliers on model results while retaining useable observations, we report results of those analyses here. Our choice of US$25,000, or the 99th percentile, is also consistent with others [15-17].

As part of model development, we added interaction terms as covariates and conducted additional analyses where we limited study subjects to employees, males, and male employees, reflecting the larger number of employees and males in the study group. To account for nonlinear effects of DASH participation and website use intensity, we grouped study year costs into quartiles; we also grouped website visits and study year costs into quartiles. There were no differences suggesting that the interaction terms, study population restrictions or groupings should be included in the final model as none of these modifications affected the results. We repeated the analyses we report here using square root and log transformations for all cost variables because of their good performance with heteroscedastic health care cost data [18]. Our results based on the transformed data were similar to those observed using the untransformed data. Thus, to allow for ease of interpretation, we present results here for the untransformed data.

## Results

### All Study Subjects

Demographic, ARI, and baseline medical and pharmacy cost information were gathered for all study subjects (N = 15,237) and for the subgroup of subjects with CV risk conditions (N = 735). These measures were also examined for DASH (N = 1967) and non-DASH (N = 13,270) participants overall and in the CV risk group (DASH: N = 134; non-DASH: N = 601). These results are shown in Table 1.

**Table 1:** Baseline characteristics of DASH and non-DASH participants

		All Study Subjects			CV Risk Subgroup^a^		
		Total (N = 15,237)	DASH Participants (N = 1967)	Non-DASH Participants (N = 13,270)	Total (N = 735)	DASH Participants (N = 134)	Non-DASH Participants (N = 601)
**Cost baseline year (US$)**							
	mean (SD)	2684 (7164)	2181 (4351)	2758 (7489)	5663 (10,089)	4239 (6335)	5980 (10,727)
	P25/P50/P75^b^	327/934/2612	345/933/2224	324/935/2563	1401/2772/5783	1002/2028/4527	1490/2849/6020
**Cost DASH year (US$)**							
	mean (SD)	2814 (7835)	2413 (4315)	2879 (8228)	5929 (13,611)	3425 (3667)	6487 (14,897)
	P25/P50/P75	358/1006/2621	442/1145/2700	347/981/2607	1394/2681/5755	1146/2318/4222	1467/2848/6152
**Baseline year ARI**							
	mean (SD)	3.70 (6.80)	3.52 (6.04)	3.72 (6.91)	12.14 (13.70)	10.10 (9.73)	12.59 (14.41)
	P25/P50/P75	1/1/3	1/1/3	1/1/3	3/7/16	2/6/14	3/8/17
**Age (years)**							
	mean (SD)	40.2 (9.2)	40.7 (9.1)	40.1 (9.2)	47.5 (8.54)	46.1 (8.28)	47.8 (8.57)
	P25/P50/P75	33/40/46	34/41/47	33/40/46	42/49/54	41/47/52	42/49/54
**Gender, % (no.)**							
	Male	46 (7041)	56 (1116)	45 (5925)	65 (476)	73 (98)	63 (378)
	Female	54 (8196)	44 (851)	55 (7345)	35 (259)	27 (36)	37 (223)
**Enrollment status, % (no.)**							
	Employee	55 (8384)	84 (1659)	51 (6725)	64 (469)	85 (114)	59 (355)
	Spouse	45 (6853)	16 (308)	45 (6853)	36 (266)	15 (20)	41 (246)
**Website visits**							
	mean (SD)	N/A^c^	12.0 (17.0)	N/A	N/A	16.9 (26.3)	N/A
	P25/P50/P75	N/A	3/9/12	N/A	N/A	3/9/17	N/A

^a^ CV risk group subjects show evidence of hyperlipidemia, hypertension, and/or diabetes in both years.

^b^ P25/P50/P75 equals 25th, 50th, and 75th percentiles.

^c^Not available.

Among the 15,237 study subjects, 55% (n = 8384) were employees, while 45% (n = 6853) were spouses. The overall study sample was 46% male (n = 7041), with an average age of 40.2 years. Average total baseline year health care costs were US$2684. A slightly higher proportion of the DASH participants was male, compared to the non-DASH participants (56% vs 45%). The average age of the DASH participants was 40.7 years, slightly higher than the non-DASH participants (40.1 years). DASH participants were mostly employees (84%; n = 1659). DASH participants had a mean ARI of 3.52, which was slightly lower than the mean ARI for the non-DASH participants (3.72). Average total costs among DASH participants in the baseline year were lower than among the non-DASH participants (US$2181 vs US$2758).

Table 1 also shows demographic, ARI, and baseline year health care costs for the DASH and non-DASH CV risk groups. This subgroup was 65% male (n = 476) and 64% employees (n = 469), with an average age of 47.5 years, older than the overall study sample. The mean baseline year ARI of 12.14 (DASH: 10.10, non-DASH: 12.59) was also higher than in the general study sample, as were mean total baseline health care costs (overall: US$5663; DASH participants: US$4239, non-DASH: US$5980).

**Table 2:** Predictors of costs in DASH year: DASH vs non-DASH (overall and CV risk group)^a^

	All Study Subjects (N = 15,237)		CV Risk Group (n = 735)	
	Difference in Mean Study Year Cost^b^ (SE)	*P* (*t*^c^)	Difference in Mean Study Year Cost^b^ (SE)	*P* (*t*^c^)
DASH use vs non-use^d^	$85.14 ($90.83)	.35 (0.94)	−$826.95 ($424.81)	.05 (1.95)
Age	$8.18 ($3.31)	.01 (2.47)	$50.40 ($25.85)	.05 (1.95)
Male vs female	−$453.05 ($156.98)	.004 (2.89)	$88.90 ($645.85)	.89 (0.14)
Employee vs non-employee	−$136.45 ($370.54)	.72 (0.37)	−$2862.85 ($1564.49)	.07 (1.83)
Baseline year ARI^e^	$123.46 ($5.92)	< .001 (20.85)	$133.69 ($17.61)	< .001 (7.59)
Baseline year cost^f^	$0.29 ($0.01)	< .001 (29.00)	$0.34 ($0.04)	< .001 (8.50)

^a^ Baseline and study year costs top-coded at US$25,000; study year website visits top-coded at 75; probability of DASH participation included as model covariate (not shown).

^b^ For age, ARI, baseline year cost: difference in mean study year costs per unit difference; unit is one year (age), one integer (ARI), one US dollar (baseline year cost).

^c^ Degrees of freedom = n − 6.

^d^ DASH participants’ health care costs were, on average, US$85 higher than those of nonparticipants, although this result was not statistically significant.

^e^ Higher baseline year ARI increases were associated with higher study year costs. On average, study year costs increased US$123 with each additional unit increase in the baseline year ARI. A unit refers to an integer; as an example, an ARI of 10 is one unit greater than an ARI of 9.

^f^ Higher baseline year health care costs were associated with higher study year costs. On average, study year costs were US$0.29 higher for each additional dollar in baseline year cost.

The results of the linear regression analysis of study year costs for DASH vs non-DASH in the full sample are shown in Table 2. Among all study subjects, DASH participation was associated with increased health care costs, although this result was not statistically significant (difference in mean costs, DASH vs non-DASH = US$85.14; *P = .*35). Model covariates associated with significantly higher study year costs were older age (*P = .*01), being female (*P = .*004), higher baseline ARI (*P < .*001), and higher baseline year costs *(P < .*001).

Results of the linear regression analysis of study year costs for DASH and non-DASH CV risk group study subjects are also shown in Table 2. DASH CV risk group members’ study year health care costs were US$827 lower, on average, than those of the non-DASH CV risk group members (*P = .*05). Higher baseline year ARI was significantly associated with higher study year costs, with each additional unit of risk associated with an increase, on average, of US$134 (*P < .*001). Each additional dollar in baseline year costs was associated with an additional US$0.34, on average, in study year costs (*P < .*001).

**Table 3:** Cost in DASH year as a function of intensity of website use, adjusting for covariates^a^

	All DASH Participants (n = 1967)		CV Risk Group (n = 134)	
	Difference in Mean Study Year Cost^b^ (SE)	*P* (*t*^c^)	Difference in Mean Study Year Cost^b^ (SE)	*P* (*t*^c^)
Change in costs per website visit^d^	−$6.66 ($5.34)	.21 (1.25)	−$28.45 ($14.61)	.054 (1.95)
Age^e^	$34.07($8.05)	< .001 (4.23)	$17.50 ($31.57)	.58 (0.55)
Male vs female	−$458.43 ($161.07)	.005 (2.85)	−$1121.62 ($686.75)	.11 (1.63)
Employee vs non-employee	$66.82 ($215.01)	.76 (0.31)	−$39.67 ($861.64)	.96 (0.05)
Baseline year ARI^f^	$60.24 ($16.74)	< .001 (3.60)	$54.48 ($36.69)	.14 (1.48)
Baseline year cost^g^	$0.30 ($0.03)	< .001 (10.00)	$0.34 ($0.07)	< .001 (4.86)
	DASH Participants Website Use at or Above Median^h^ (n = 1028)		CV Risk Group Website Use at or Above Median^h^ (n = 80)	
	Difference in Mean Study Year Cost^b^ (SE)	*P* (t^d^)	Difference in Mean Study Year Cost^b^ (SE)	*P* (t^d^)
Change in costs per website visit	−$14.26 ($6.97)	.04 (2.05)	−$54.61 ($20.16)	.01 (2.71)
Age	$49.22 ($11.87)	< .001 (4.15)	$18.69 ($47.47)	.70 (0.39)
Male vs female	−$444.33 ($230.67)	.05 (1.93)	−$1855.55 ($940.40)	.05 (1.97)
Employee vs non-employee	$141.08 ($301.12)	.64 (0.47)	$757.00 ($1286.59)	.56 (0.59)
Baseline year ARI	$109.73 ($26.49)	< .001 (4.14)	$98.59 ($55.97)	.08 (1.76)
Baseline year cost	$0.24 ($0.04)	< .001 (6.00)	$0.25 ($0.12)	.04 (2.08)

^a^ Baseline and study year costs top-coded at US$25,000; study year website visits top-coded at 75.

^b^ For number of website visits, age, ARI, baseline year costs: difference in mean study year costs per unit difference; unit is one year (age), one integer (ARI), one US dollar (baseline year cost).

^c^ Degrees of freedom = n − 6.

^d^ Among all DASH participants, each additional website visit was associated, on average, with a US$6.66 decrease in study year health care cost. This result was not statistically significant. Among CV risk group DASH participants, each additional website visit was associated with a US$28 decrease in study year cost; this result was not statistically significant at the *P* < .05 level. Among DASH participants who visited the website at least the median number of times during the study year (nine visits), each additional visit was associated with a US$14 study year cost decrease. Among CV risk group DASH participants who visited the website at least the median number of times for the CV risk group (also nine visits), each additional visit was associated with a US$55 decrease in study year cost.

^e^Among all DASH participants, each additional year of age was associated with US$34, on average, higher study year health care cost. Among DASH participants who visited the website nine or more times during the study year, each additional year of age was associated with US$49 higher study year health care cost. Among CV risk group DASH participants, the relationship of age to study year health care costs was not statistically significant at the *P* < .05 level.

^f^Among all DASH participants, each additional increment in baseline year ARI was associated with US$60 higher study year health care cost.

^g^ Each additional dollar in baseline year costs was associated with increased study year health care costs as follows: US$0.30 among all DASH participants; US$0.34 among CV risk group DASH participants; US$0.24 among DASH participants who visited the website at least nine times; US$0.25 among CV risk group DASH participants who visited the website at least nine times.

^h^ Median website usage for all DASH and CV risk group DASHparticipants: nine visits.

Results of the analyses of intensity of website usage and study year costs are shown in Table 3. Among 1967 DASH participants, each additional website visit was associated with almost a US$7 decrease in study year health care costs, but this result was not statistically significant (*P = .*21). Among participants whose website usage was at or above the nine-visit study year median (n = 1028), each additional website visit was associated with a US$14 decrease in health care costs on average (*P* = .04).

**Figure 3:**
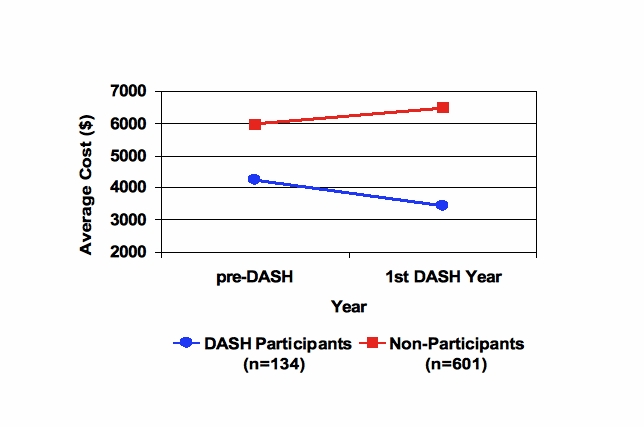
CV risk group change in unadjusted total costs from baseline to DASH study year in DASH participants vs nonparticipants

Figure 3 compares the change in crude study year costs, relative to baseline year costs, among DASH participants versus nonparticipants in the CV risk group. Among DASH CV risk group participants, study year health care costs were US$814 lower than baseline year costs. Among DASH nonparticipants, study year costs were US$507 higher than baseline year costs. These results are not adjusted to control for age, gender, employee status, or baseline ARI.

Results of the analysis of website use intensity in the DASH CV risk group are also shown in Table 3. Among the 134 DASH CV risk group members, each additional website visit was associated with a decrease of approximately US$28 in health care costs (*P = .*05). Among those who used the website at least the median number of nine times during the year (n = 80), each additional visit was associated with a US$55 decrease in study year costs (*P = .*01).

## Discussion

We did not find DASH participation to be associated with lower health care costs in the overall study sample. With an average age of 40 years, an average ARI of 3.7, and minimal health services utilization, this study sample is relatively young and healthy. The significance of higher baseline year risk and costs, female gender, and increasing age as predictors of study year costs is consistent with a relatively young, commercially insured population. Improvements in diet and exercise habits would not be expected to address the highest expenditures in this group—pregnancy and childbirth, depression, and back pain.

We found suggestive evidence that DASH program use was associated with decreased health care spending among study subjects with CV risk. Within the DASH CV risk group, participants’ study year health care costs were US$827 lower than for the DASH nonparticipants. Although this result only achieved a significance level of *P* = .05, the reduction in mean costs from DASH participation (US$827), which was nearly twice the standard deviation (US$425), indicates that the effect size is quite large; the model R-square, 39%, is also noteworthy. Improvements in diet and exercise would be expected to address health care expenditures among study subjects with hyperlipidemia, hypertension, or diabetes. This subgroup is older (average age 47 years) and less healthy, with a baseline year ARI more than three times that of the general study sample.

We also found evidence suggestive of a dose-response relationship. Among DASH participants who visited the website at least nine times during the study year, each additional visit was associated with lower study year costs overall (*P = .*04) and in the DASH CV risk group (*P* = .01). Evidence of this dose-response was strongest among the DASH participants in the CV risk group, where each additional website visit was associated with a US$55 decrease in study year health care costs (*P = .*01).

The DASH CV risk group participants’ baseline ARI (10.10) suggests that they may be less sick than the non-DASH CV risk group (baseline ARI = 12.59). However, the DASH CV risk group, with conditions targeted by the DASH program, was the only subgroup that showed a decrease in health spending. Health care costs among DASH enrollees decreased 24% between the baseline (US$4239) and study years (US$3425) in the DASH CV risk group. By contrast, health spending increased US$507 (8%) in the non-DASH CV risk group and approximately US$130 per person (4.8%) across the general study sample.

We also found evidence of selective enrollment in the DASH program by study subjects with hypertension, hyperlipidemia, and diabetes. Despite being offered as a benefit to all employees and their dependents, participation in DASH for Health was proportionally higher (18%) among individuals with CV risk conditions (ie, those who might benefit most from nutrition improvement) than it was among individuals without those conditions, of whom 13% signed up for the DASH program (*P* < .001). For employers interested in offering benefit programs equally to all employees rather than targeting a selected subset of the employee population, this finding provides evidence that the DASH for Health program benefits the subset of the employee population whose health risks are of concern and whose health status is targeted by the program.

Our results expand on the already-published reports that Internet-based programs can have positive effects on clinical parameters such as weight and blood pressure [11,19-22] and contribute to our understanding of the effects of nutrition, weight management, and exercise programs on health care costs in targeted populations [23-26], including workplace populations [3,27-32]. Several recent studies indicate that workplace-sponsored, Web-based programs can lead to these improvements in clinical parameters [11,33-36]. Our focus on the effects of an employer-sponsored, Web-based diet and exercise program on health care costs also expands our understanding of the effects of employer benefits that encourage employees to better manage their health status and contain health care costs. Hsu et al [37] observed significant increases in nonpharmaceutical health care expenses over a short time period among persons with chronic conditions who reduced their prescription medication use in response to increased cost sharing in benefit design. By contrast, regular use of the DASH for Health program may encourage health behavior changes that result in cost savings among persons with chronic conditions in a similarly short time frame. The benefits of DASH participation among healthier, younger enrollees may be evident over a longer time period than the year evaluated for this study. Employers, particularly those who are self-insured, may be interested in both short- and long-term employee costs and health status and choose to invest in health status improvements that will show benefits over the long term [38-40].

### Limitations

A number of study limitations should be noted. Our analysis used observational data and is vulnerable to selection effects, which represent the largest threat to validity in observational studies. Our use of a control group and a pre-test, coupled with our use of a propensity score, are standard mechanisms for addressing selection bias in quasi-experimental study designs. However, our model would not control for the possibility that study subjects who were motivated to manage their CV risk conditions were more likely to enroll in DASH for Health and would visit the website more often. However, it is unlikely that our finding that even moderate use of the DASH for Health program is associated with lower health care costs is attributable to motivation and not to participation in the program. This finding is consistent with clinically oriented studies of the DASH diet program indicating that the DASH diet is associated with improved blood pressure, lower cholesterol levels, and increased insulin sensitivity. Such clinical improvements would be expected to be associated with lower health care costs.

Several other limitations should be noted. First, the study sample was limited to persons with health care costs in both years. The proportion of study subjects with costs in only one year (costs in baseline year only: 90%; costs in study year only: 79%) is consistent with the national Medical Expenditure and Panel Survey (MEPS) data indicating that 11% of commercially insured persons do not use health services in a given year. D2Hawkeye received and cleaned the source claims data and reported that the proportion of study subjects with zero costs was higher than expected. Because the data could not be sent back to the data warehouse for review, we chose to restrict the study to persons with costs in both years. In analyses with all study subjects, including those with no costs in either or both years, costs for all DASH participants were significantly lower than for non-DASH participants, and the cost savings were higher among DASH participants with CV risk conditions. (Results available on request.) Restricting study subjects to those with costs understates the effects of the DASH program on costs. Second, our analysis compares study subjects who visited the DASH website at least once with those who did not visit it at all. However, subjects who visited the website once during the study year would not be expected to benefit from the nutrition and exercise education that the DASH for Health website program offers. Our finding of a dose-response effect would be strengthened by the inclusion of subjects who signed up for the program but never visited the website. From the perspective of benefit design, this effect would be more useful for guiding planning or coaching efforts. Third, our propensity score is based only on available data and therefore may not completely address potential selection bias. It is possible that DASH participants with CV risk conditions would have had decreased health care costs without participation in DASH. However, the clinically observable effects of the DASH diet on CV risk conditions make this result less likely. Fourth, our results are based on all health care costs for all conditions. Costs for conditions targeted by the DASH diet cannot be distinguished from costs for medical conditions that are not likely to benefit from improved diet, such as pregnancy and childbirth and back pain. However, the dose-response effect among participants who use the DASH website more often and the increasing significance of additional website usage in the DASH CV risk group suggest that participation in the DASH program is related to decreases in health care costs among persons at higher risk for health care expenses. We performed additional analyses of the effects of DASH participation on study year costs for subjects with different baseline year risk levels. Based on the regression model, at higher levels of baseline year risk, DASH participants had significantly lower study year costs than nonparticipants. (Results available on request.) These results are of particular interest given that higher levels of baseline year risk are predictive of higher costs [13]. Finally, study year costs include health care costs incurred during the initial enrollment period. The effects of DASH for Health program participation would not expected to be evident during the initial launch of the program or during the first few months of the year. However, this limitation suggests that our study results understate the effects of DASH for Health program participation since they include this initial time frame.

Our focus on short-term cost savings does not examine whether these savings are offset by increases in other costs [40]. Although the DASH for Health program is relatively inexpensive, further study to evaluate its cost-effectiveness as a corporate benefit would be beneficial. Finally, the study sample was limited to employees at one technology firm and their dependents. Our study results may not be generalizable to persons without employer-based insurance or to adults who are less comfortable using the Internet for information about diet, exercise, and health management. Research into the effectiveness of DASH for Healthin other populations is warranted.

### Conclusions

Use of an Internet-based program that targets changes in diet and exercise to reduce weight, cholesterol, and blood pressure shows evidence of reducing short-term health care costs among persons at high risk for health care expenditures with conditions targeted by the diet. Offering access to a website with diet and exercise information appears to have promise as a low-cost, employer-sponsored benefit that contributes to lower health care costs among persons at higher risk for above-average health costs and utilization.

## Abbreviations

ARI: adjusted risk index
BUSM: Boston University School of Medicine
CV: cardiovascular
DASH: Dietary Approaches to Stop Hypertension
